# Social groupwork for promoting psychological well-being of adolescents enrolled in sponsorship programs

**DOI:** 10.12688/f1000research.52532.1

**Published:** 2021-06-30

**Authors:** Shinto Joseph, Dr. Sheeja Remani B Karalam

**Affiliations:** 1 Department of Sociology and Social Work, CHRIST (Deemed to be) University, Bengaluru, Karnataka, 560029, India

**Keywords:** Intervention Research, social group work, marginalized adolescents, psychological well-being, empowerment

## Abstract

**Background: **The dearth of data on adolescents highlighted in the UN’s data disaggregation against the agenda ‘no one left behind’ calls for research on ‘the second decade’. Moreover, India is a country with the world’s largest adolescent population, and as such, studies and policies for developing competencies of adolescents are crucial to the country’s development; interventions instilling confidence to aspire to a better future in underprivileged adolescents are vital to mitigate inequity.

**Methods: **This intervention study adopted a quasi-experimental design to measure the effectiveness of social groupwork in raising the psychological well-being of adolescents in child sponsorship programs in Kerala. Forty adolescents from a Child Sponsorship Program (CSP) center in Kochi were recruited for the study. Those suggested by the CSP center considering their poor academic performance and behavior problems were allocated to the intervention group and the rest to the comparison group.  The intervention was designed in response to the information garnered through a preliminary study and administered to the intervention group (n=20). We conducted pre-test and post-test for both the intervention group and comparison group (n=20).

**Results: **Comparison between pre- and post-measurements carried out using paired sample t-test for the intervention group and comparison group separately gave a p-value of <0.05 for the intervention group and >0.05 for the comparison group. Thus, it was proved that psychological well-being of participants in the intervention group was raised significantly due to the social group work intervention.

**Conclusions: **Applying refined granularity, this research adds data specifically on adolescents enrolled in child sponsorship programs and sets a blueprint for social groupwork to improve their psychological well-being. Proposing a conceptual framework for child sponsorship programs, this study recommends further research in all aspects of its functioning, and interventions at group, family, and community levels, for the well-being and empowerment of marginalized adolescents.

## Introduction

Assigning a “combined focus on the individual and the society” ( Alissi, 1982:10), social group work (SGW) stands out as a quintessential method in the social work domain. SGW is developed to assist individuals as well as the group in meeting their needs and solving their problems through group experiences enabled by group workers ( Benson, 2001; Konopka, 1983; Lindsay and Orton, 2014). Facilitating “learning from each other” ( Teater, 2014:86), SGW affirms individual’s strengths and encourages mutual aid ( Gitterman and Salmon, 2009). In contrast to methods which are primarily therapeutic, SGW engages in enhancing behavioral, emotional, and social well-being of individuals through group processes ( Zastrow, 2015).

The core tenets of social work are enrichment of psychosocial functioning of humans and enhancement of their living environments ( Northen and Kurland, 2001). Therefore, social workers are primarily concerned with specialized intervention accomplishing well-being of individuals. Since human needs and satisfaction vary in the flux of time, the concept of well-being assimilates new constructs while redefining the existing ones. A broad classification of well-being assigns it into two categories: internal or subjective well-being (SWB), and external or objective well-being (OWB) ( Alatartseva and Barysheva, 2015; Schueller and Seligman, 2010). The latter defines well-being in terms of social indicators such as accessibility to resources and social networks, and attainability of food, shelter, education, employment, and health care ( Schueller and Seligman, 2010; Western, Tomaszewski and Zeeb, 2016), whereas the former conceptualizes the notion of well-being on different philosophical frameworks and redesigns the models, incorporating nuances evolved through scientific research. Two predominant models of SWB are Diener’s (1984) hedonia model expressed by communicative emotions, and Ryff’s (1989) eudaimonia model concerning the inner experience of contentment and resilience. However, by involving a goal-directedness towards personal growth, tapping one’s potential, Ryff’s model of psychological well-being (PWB) gains distinctiveness ( Disabato, *et al*., 2016). An extant investigation into well-being trajectories; “pleasure, engagement and meaning, suggests that engaging and meaningful activities may have stronger influences on well-being than pursuing pleasure” ( Schueller and Seligman, 2010:253). These conceptions second researchers’ stand in adopting Ryff’s PWB model for intervention studies with specific objectives such as finding true self, gaining mastery over environment, creating meaningful relationships, achieving autonomy, finding meaning in life, and attaining personal growth in promoting one’s well-being ( Schlegel *et al.*, 2009). Ryff’s revised PWB model probes into six dimensions of well-being:

(1) the extent to which respondents felt their lives had meaning, purpose and direction (purpose in life); (2) whether they viewed themselves to be living in accord with their own personal convictions (autonomy); (3) the extent to which they were making use of their personal talents and potential (personal growth); (4) how well they were managing their life situations (environmental mastery); (5) the depth of connection they had in ties with significant others (positive relationships) and (6) the knowledge and acceptance they had of themselves, including awareness of personal limitations (self-acceptance) ( Ryff, 2014:11)

Adolescence is a “stormy developmental stage” ( Zastrow, 2015, p.450) characterized with emotional upheaval associated with physical, cognitive, psychological, and social growth as adolescents internalizes many traits of emotional instability ( Asendorpf and Van Aken, 2003; Rosenberg, 1979). The capabilities that adolescents acquire serve as a base for their well-being through their life; conversely, their failure in acquiring capabilities in adolescence adversely affects not only their personal life, but their family and community as well ( Sheehan *et al*., 2017). A heuristic model that correlates psychosocial functioning of adolescents with the supportive direction of groups, relationships, and interactions, exemplifies the role of SGW in fostering PWB ( van Aken *et al.*, 1999).

Studies among adolescents posit adverse effects of social stratification on their developmental outcomes ( Benner *et al.*, 2018; Wheeler *et al.*, 2020), which mandates social workers’ attention on adolescents from lower socio-economic strata or low-income families. While child sponsorship programs by international, national, and local sponsoring agencies and organizations improve OWB of adolescence by enabling accessibility to education and employment, their SWB remains under-researched ( Wydick *et al.,* 2013). There exists a correlation between children’s psychosocial competencies and material poverty of their family, so that the adolescent development dimensions such as self-efficacy, self-esteem, sense of inclusion, and educational aspirations score lower than that of children with a privileged family environment (Dercon and Krishnan, 2009). Studies from economic streams draw the conclusion that the poverty trap originates from internal constraints such as failure to aspire, lack of self-efficacy, and absence of hope, rather than external constraints ( Bernard *et al.,* 2011; Dalton *et al.,* 2010). The strategies that they put forth for social mobility of people from lower strata by raising their aspirations are; behavior modification, observing role models, choosing cognitive windows to look at people with higher levels of self-confidence, and establishing social links to transfer aspirations. Heeding these scientific study recommendations, a few sponsoring agencies initiated after-school programs aiming at the raising aspirations and self-esteem of students ( Glewwe *et al.*, 2014; Wydick *et al.,* 2013).

The strategic move of sponsorship agencies to nurture PWB of students provides an opening for social workers to work with groups. Concomitant research in SGW is imperative owing to paucity of research in this area ( Breton, 2006). Research in social work involves understanding the problems that people are experiencing within society, and the effect and implications of social policies and expert interventions on individuals and society as well ( Wilson *et al.,* 2008). Regardless of the growing attention, there exists a dearth of intervention research in social work ( Mishna *et al.,* 2012). In the study outlined in this article, we analyzed the impact of an SGW intervention on adolescents enrolled in a sponsorship program by measuring their PWB statistically. Agile construction and administration of SGW tools targeting enhancement of PWB of participants brought about the desired outcome and created a framework for SGW with adolescents enrolled in sponsorship programs.

### Philosophical assumptions

Espousing a transformative world view ( Mertens, 2008; Creswell and Creswell, 2017) we designed an interventional study for uplifting the PWB of a socially marginalized group of adolescents from low-income families. A transformative paradigm facilitates change in the lives of participants, addressing specific issues such as inequality, oppression, and alienation. In this study, our focal point was social stratification perpetuating the low PWB of adolescents. The SGW intervention was tailored to raise participants’ PWB to make them feel equal to their counterparts from more advantaged stratas of society.

### Theoretical framework

The theoretical conceptualization of an intervention research study involves two stages: delineation of a problem theory, and of a program theory ( Fraser and Galinsky, 2010). Delineation of a problem theory provides a clear understanding of the psycho-social conditions that produce problems, whereas, program theory underpins the intervention for achieving expected outcome.

With a person-in-environment perspective ( Hare, 2004; Weiss-Gal, 2008), this study comprehended the psycho-social problems of adolescents induced by the low-income family environment. Ryff’s six-factor PWB model ( Ryff, 2014, 1995), which combines different perspectives such as ‘fully functioning person’ ( Rogers, 1961:183), ‘executive processes of personality’ ( Neugarten, 1973:312), ‘self-actualization’ ( Maslow, 1968), ‘individuation’ (Jung, 1958), ‘will to meaning’ (Frankl,1959), ‘personal development’ ( Erikson, 1959), ‘basic life tendencies’ (Buhler,1935) and ‘maturity’ ( Allport, 1961), provided a theoretical base for our SGW intervention program to augment the PSW of participants. The group was formed within the framework of empowerment theory ( Zimmerman, 2000) in general, and particularly its personal construct, psychological empowerment (PE) ( Zimmerman, 1995; Zimmerman *et al.*, 1992).

## Methods

This research was conducted with a general objective to raise the PWB of adolescents in sponsorship programs, and specific objectives; i) to raise autonomy of adolescents in sponsorship programs, ii) to raise environmental mastery of adolescents in sponsorship programs, iii) to raise personal growth of adolescents in sponsorship programs, iv) to raise positive relations of adolescents in sponsorship programs, v) to raise purpose in life of adolescents in sponsorship programs, vi) to raise self-acceptance of adolescents in sponsorship programs. Alternative hypothesis set was; H( *a*): There is a significant difference between the PWB levels of participants before and after the SWG intervention. Effect of SGW intervention on all the six components of PWB were tested separately against hypotheses; *H(1)* : There is a significant difference between autonomy of participants before and after the SGW intervention; *H(2)* : There is a significant difference between environmental mastery of participants before and after the SGW intervention; *H(3)* : There is a significant difference between personal growth of participants before and after the SGW intervention; *H(4)* : There is a significant difference between positive relations of participants before and after the SGW intervention; *H(5)* : There is a significant difference between purpose in life of participants before and after the SGW intervention; *H(6)* : There is a significant difference between self-acceptance of participants before and after the SGW intervention.

### Ethical statement

This study was approved by the Center for Research of the CHRIST (Deemed to be) University, Bengaluru, which constitutes the Institutional Review Board/Ethics Committee that approved this study, (approval number: CU:RCEC/00037/l/19).

The Child Sponsorship Program (CSP) of Rajagiri OutREACH gave consent to conduct this study and the same was approved by the Institutional Review Board/Ethics Committee of CHRIST (Deemed to be) University. Objectives of intervention were set in line with visions and missions of the center. The purpose of the study was well explained and written informed consent for participation and publication data was obtained from participants and their parents, prior to the study. Pseudonyms were assigned to all participants for deidentification in the recording and reporting.

### Trial registration

This trial is registered at the Centre for Research/IRB of CHRIST (deemed to be) University. The trial registration number is CU.CFR.PhD.CWCL.REG.No.1730087.2017.

### Research design

Our research design focused on the research question ‘is SGW effective in improving PSW of adolescents in sponsorship programs?’ ( Kate, *et al.,* 2018). We adopted a quasi-experimental, non- randomized control group design ( Campbell and Stanley, 1963; Creswell and Creswell, 2017; Shadish *et al.*, 2001) for the study. In this design both the intervention group (IG) and the comparison group (CG) took pre-test and post-test for measuring PWB before and after intervention while only the IG received the SGW intervention for raising their PWB. Inferences were made comparing scores of both groups.

A shorthand notation for this design is:

**Table:** 

I	Y1	X	Y2
C	Y1	Non-X	Y2

I - intervention group;

C - comparison group;

Y1 - measurement of PWB in pre-test;

Y2 - measurement of PWB in post-test;

X - social groupwork (intervention).

### Sponsoring agency

Rajagiri OutREACH, a professional service wing of Rajagiri College of Social Sciences, Kochi, reaches out to the community at large through various programs including the child sponsorship program (CSP). Across the state of Kerala, there are 30 CSP centers of Rajagiri OutREACH, which mainly provide economic support to children (11-18 years) from lower socio-economic stratas, and promote studies and services aiming at uplifting marginalized children. Currently, there are 2,862 children enrolled with Rajagiri OutREACH, and among them 1,086 belong to the13-16 years age group.

### Research setting

From the 16 total CSP centers in Kochi run by Rajagiri OutREACH, one center at Kalamassery was selected as the study setting with two criteria; first, the study excluded the centers with less than 40 adolescents in them, and second, one center was selected using random sampling ( Levy and Lemeshow, 2013); by assigning unique numbers to each center and randomly picking one from them. Entry to and consent for conducting this study at the center were negotiated through proper channels ( Høyland *et al.*, 2015). Recruitment, intervention, and data collection with participants were done in this CSP center. The study was conducted over a period of two years from June 2018 to July 2020.

### Preliminary study

Secondary data was garnered from the selected CSP center records that revealed the poor academic performance, behavior problems, and issue coping with family of registered adolescents. For an in-depth exploration of the problems of participants and for identifying risk factors and protective factors, we employed case study as a method of inquiry ( Yin, 2018), and conducted twenty case studies among participants and their parents, with prior consent and appointment.

### Delphi survey

**Figure 1.**
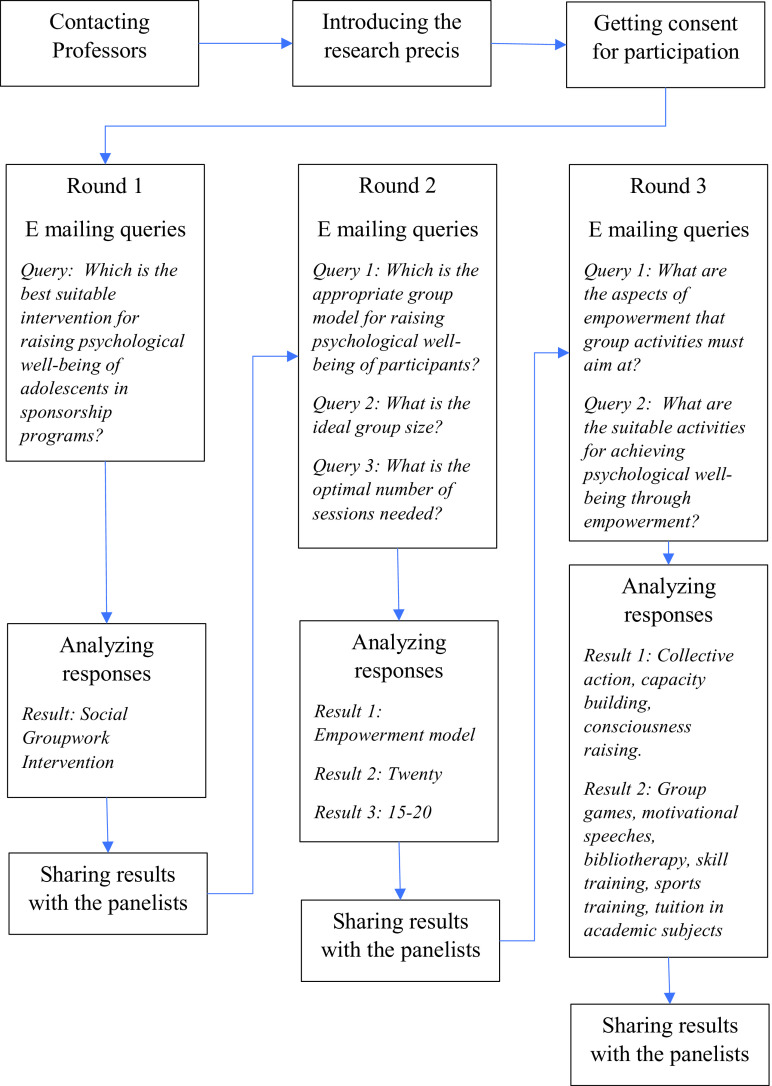
Delphi survey process and results.

Based on the conclusion drawn from the preliminary study the first author under the guidance of second author developed questionnaires and conducted a ‘Delphi survey’ (Hasson *et al.*,2000) among selected (purposive sampling) social work professors in South India. We e-mailed professors in three leading social work colleges in South India at Rajagiri College, Kalamassery, Marian College, Kuttikkanam and Christu Jayanthi College, Bangaluru, and nine out of the fifteen we contacted expressed their willingness for participation in the survey by returning the electronically signed consent form which was attached in the mail. The panelists reached consensus after three rounds, on the most suitable method to enhance the psychological well-being of adolescence enrolled with CSP (See Figure 1 for details).

**Figure 2.**
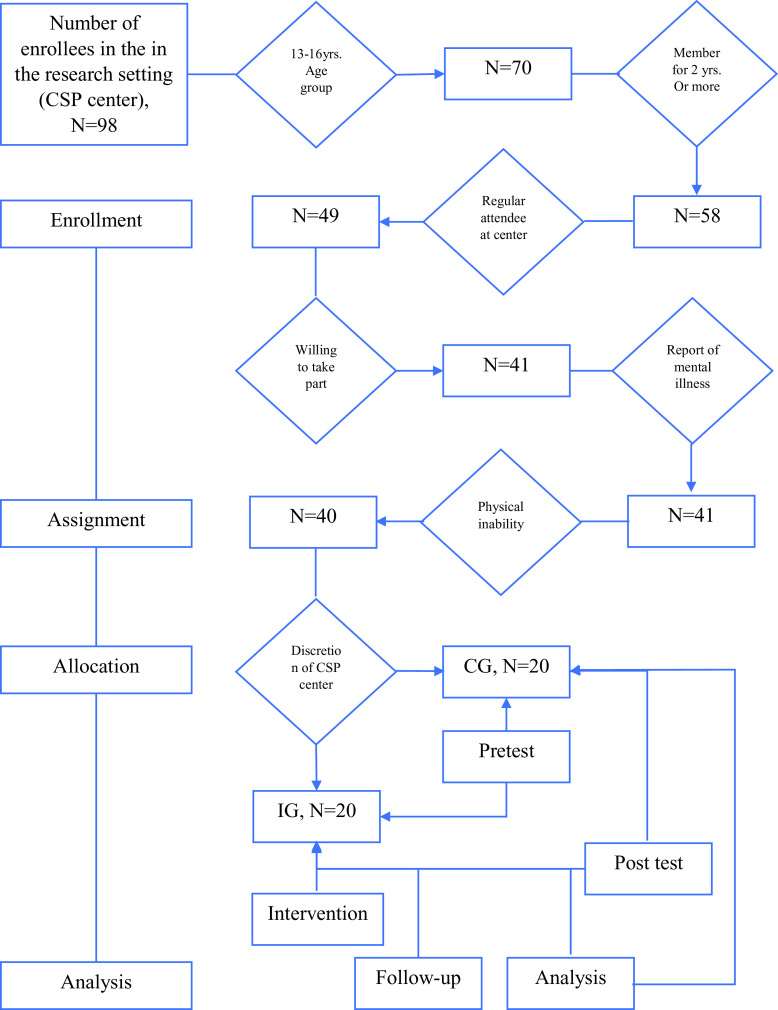
Participant flow.

**Table 1.** Intervention schedule.

Session No:	Duration	Activity	Expected outcome	Resource person	Setting
1	2 hrs.	Self-introduction, Group games	Group familiarity, Group interaction	Researcher/Groupworker	CSP center hall
2	2 hrs.	Explaining group objectives, Group games	Reducing anxiety, Orientation to the purpose of program	Researcher/Groupworker	CSP center hall
3	2 hrs.	Setting norms, Group games	Mutual respect, Mutual trust	Researcher/Groupworker	CSP center hall
4	2 hrs.	Showcasing talents, Assigning roles	Self- respect, Accountability	Researcher/Groupworker	CSP center hall
5	2 hrs.	Bibliotherapy	Knowing successful personalities, Self-acceptance, Feeling worthy	Student counsellor	CSP center hall
6	2 hrs.	Motivational speaking	Confidence and hope for a better future	Preacher	CSP center hall
7	2 hrs.	Life goal setting	Ambition	Researcher/Groupworker	CSP center hall
8	2 hrs.	Seminar on importance of education Seminar on drug abuse and consequences	Cognizant of the need for studying well Aversion to drugs	Social work educator	CSP center hall
9	2 hrs.	Football training Swimming training	Personal skills, Group cohesion	Football coach Swimming trainer	CSP center ground CSP center pool
10	2 hrs.	Lecture on rights and equality Lecture on personal integrity	Awareness on rights Awareness on values	Social work educator	CSP center hall
11	2 hrs.	Vegetable gardening	Personal skill, Family support	Field officer	CSP center backyard
12	2 hrs.	Tuition in English	Improved academic performance	Subject teacher	CSP center hall
13	2 hrs.	Tuition in mathematics	Improved academic performance	Subject teacher	CSP center hall
14	2 hrs.	Tuition in Science	Improved academic performance	Subject teacher	CSP center hall
15	2 hrs.	Peer learning	Peer support in learning	Researcher/Groupworker	CSP center hall
16	2 hrs.	Recapitulation of sessions, reflections, winding up	Acknowledging and stabilizing the gains of intervention	Researcher/Groupworker	CSP center hall
*Research setting is the CSP center at Kalamassery, Kochi*					

### Sampling

We did a two-stage sampling for this study. 1) Forty adolescents from the selected CSP center were selected for the study using the following inclusion/exclusion criteria.

*Inclusion criteria*

• Adolescents (13-16 years.) with the sponsorship program for two years or more.
• Regular attendance at the center
• Willing to participate in the study

*Exclusion criteria*

• Report of mental illness
• Physical inability to be involved in activities

The pretest was conducted for all the 40 participants.

2) Delphi survey results clearly stated that the ideal group size for SGW intervention is twenty. Therefore, twenty adolescents suggested by the CSP center considering the urgency of a solution to their problems such as poor academic performance and behavior problems, were assigned to the IG and the rest to CG, for ethical reasons outlined in the blinding section that the objectives of a SGW must go in line with that of the research settings, which in turn assist them to better handle the issues they are currently dealing with.

### Blinding

We found that blinding of intervention to IG is impossible in SGW intervention as the objectives of group has to be described to group members in the group forming stage itself. Blinding of researcher was also impossible in this study since the researcher was administering the intervention which was developed by him. However, data collection was done by blinded research assistants and all participants were anonymized during data analysis. Further, the bilingual translators were also blinded in this study. This research kept CG unaware of the study objectives to reduce the risk of bias.

### Tools for data collection

Socio-demographic details of participants were collected using a self-administered questionnaire ( Joseph and Karalam, 2021). The 42-item version of Ryff’s PWB scale was administered for pre- and post-test, for collecting statistical data and estimating the effectiveness of intervention in raising PWB of participants. The scale is a six-point Likert scale ranging from strongly disagree to strongly agree. Ryff’s PSW scale has six subscales which measure “autonomy, environmental mastery, personal growth, positive relations, purpose in life, and self-acceptance” ( Gao and McLellan, 2018:2). All items were translated into Malayalam (local language) and back translated into English once completed, ruling out discrepancies. The translation was done by a bilingual person and the accuracy is checked by the researcher. Pretesting was done by administering the scale to five adolescents and internal consistency was calculated statistically for each subscale after reversing the negatively phrased items. The average value obtained for Cronbach alpha coefficients was 0.79, which is > 0.7, indicating reliability of scale ( Pallant, 2020).

Secondary outcomes such as improvement in academic performance, personal competence and social competence were measured through follow up, which was carried out using in-depth interviewing of parent (father/mother) of each participant received intervention. In addition, three FGD’s were conducted with teachers of three local schools that participants were attending.

### Data collection

Socio-demographic data collection and pretests were done immediately after the selection process. Socio-demographic questionnaire and translated version of Ryff’s 42 item PWB scale (Joseph and Karalam, 2021) were distributed to participants, one after the other. Research assistants facilitated data collection which was taken place at the research setting. As it was done in the pretest, post-test to both IG and CG for assessing their PWB after the intervention was carried out in the research setting. In the preliminary study, qualitative data was collected using case study method in which the participants and parents were interviewed by the researcher at their dwellings. Secondary data was collected through reviewing records in the CSP center. Qualitative data on intervention was gathered by audio recording the sessions and participant reflections, noting down participant observations, writing researcher reflections, and collecting vignettes of group experience from participants. Follow-up interviews with parents were conducted at their houses and FGD’s in the respective schools.

### Data analysis

All the quantitative data collected was analyzed statistically using IBM SPSS statistics 25, keeping a group as the smallest unit of analysis. Paired sample t-test was employed to test hypotheses. Concurrently, qualitative data was analyzed at individual level since the effect of a SGW needs to assessed at both group and individual level. Qualitative data was transcribed verbatim and translated from Malayalam into the target language English by a bilingual expert and tested accuracy using back translation of three transcripts. Content analysis of qualitative data was done using Atlas.ti8. We did not face an issue of missing data since we could keep data collection intact with the small sample size.

### Social group work intervention

A social work intervention entails designing, testing, refining, administering, evaluating, and disseminating of a customized package. In this study we designed a social groupwork intervention in response to the concerns and needs assessed through the preliminary study among the selected adolescents enrolled in CSP, their family, and the sponsoring agency. The PE perspective contoured the groupwork program where PE refers to “empowerment at the individual level of analysis” ( Zimmerman, 1995:581). The specifically designed SGW intervention was administered to the IG.

### Purpose

The primary purpose of the social groupwork intervention was ‘enhancement of PWB of group members.’ The expected secondary outcomes of the intervention were set as improved i) personal competence, ii) academic competence, and iii) social competence of participants, bearing in mind the fact that the group objectives and activities “must be compatible with the mission and purpose of the sponsoring organization” ( Gitterman and Salmon, 2009, p.93).

### Duration

This SGW intervention was carried out in 16 sessions with a duration of two hours/session, over a period of two months.

### Group model

Placing the locus of intervention at psychological upliftment of adolescents from lower socio-economic strata, we selected the empowerment model of social groups. “The empowerment model evolved in response to the plights of marginalized and oppressed individuals of our society” ( Gitterman and Salmon, 2009:73). The empowerment model adapts a strengths perspective (( Zastrow, 2015) to counter helplessness and hopelessness. “A strengths-based approach operates on the assumption that people have strengths and resources for their own empowerment” ( Pulla, 2012:3). The significance of PE surfaces here, as it holds intrapersonal, interactional, and behavioral aspects (Zimmerman,1995). In this SGW intervention study, we framed the group activities in line with the three strategies of empowerment: collective action, consciousness raising, and capacity building.

Collective action (CA) is a joint action by a group of individuals in the pursuit of enriching their lives. Personal empowerment remains impossible in the absence of collective action for marginalized groups because they need a collective empowerment as a deprived section of the society ( Cislaghi *et al*., 2016). These SGW intervention activities were framed around the ideology of “collective action for a collective solution” (Hanish, 2000:114). The IG of adolescents from the disadvantaged strata of society was formed to act together to envision a better future, assuring their social mobility. Every group activity focused on instilling an altered perception of self; a transformation with an enlightened beliefs and attitudes in articulating aspirations.

Consciousness raising (CR) is the centrality of group process with marginalized groups ( Bruley, 2013). CR activates introspection to examine the extent of suppression within individuals and aids them to relate with others so that they can comprehend the reasons for their oppression and can think of ways to step out of it as a group. Inspiring stories of successful personalities from the same social strata were narrated for encouraging participants to dream high and to make resolutions for a bright future. In addition, an awareness on the constitutional rights, concepts of equality, and a basic lesson on the laws of the residing country was provided to implant hopes in them to set life goals, and to lessen vulnerabilities.

Capacity building (CB) is the key strategy to alleviate social disparities through empowering the weaker section ( Kaplan, 2000). This SGW intervention formulated activities to assist participants in their studies. Special tuition for those who had difficulty with particular subjects was arranged and peer learning promoted with members good at certain subjects. Training in sports and other leisure activities were also part of this intervention program. In addition, a session on kitchen gardening was organized to capacitate them in producing vegetables on their own.

### Group development

We adopted a four-stage model of group development proposed by “Northen and Kurland” ( Zastrow, 2015:22; Northen and Kurland, 2001), which highlights socio-emotional themes: a) inclusion–orientation, b) uncertainty–exploration, c) mutuality–goal achievement, and d) separation–termination, Activities grounded on the requirements of the group assisted group development through the stages towards realization of its goals ( Lindsay and Orton, 2014).

Inclusion–orientation

Post intake of members, the intervention sessions commenced as scheduled. Icebreaking techniques gave a head start by opening up the self-introduction of members, which helped them to get acquainted with other members, to reduce anxiety, and to facilitate group interaction. The GWR elucidated the objectives of the group; have faith in self, make academic progress, articulate aspirations, avoid vulnerabilities, improve familial and social relationships, and strive for a bright future.

Gaining group members’ trust was a major task for GWR. People from disadvantaged groups have enough reasons to distrust a GWR from upper stratas because of the presence of overt as well as covert discrimination in society ( Northern and Kurland, 2001). Due to this, we demystified the SGW intervention, explaining it is aimed at their well-being and empowerment. We deliberately maintained sensitivity to their feelings, treated them with respect, and behaved courteously by shaking hands, greeting warmly etc.

Interdependence is imperative in a group as it steers interactions, leading to the formation of group behavior ( Johnson and Johnson, 2005). Group games demand interdependence, which catalyzes reciprocal interactions ( Evans *et al.,* 2012). We conducted group games such as hula hoop games, relay races, and scavenger hunts to expediate ‘group cohesion’ ( Carron and Brawley, 2012). We awarded prizes for enticing members to participate actively and for motivating them to continue in the group.

We considered developing group norms in fostering helping behavior among members ( Gonzalez-Mulé *et al.*, 2014), and we shared certain norms such as mutual support, mutual respect, respect to privacy, self- disclosure stays harmless, give every task a try, it is alright to fail the tasks, open communication, conflict constructively, commitment to group and procedural norms.

Uncertainty–exploration

The first theme here is “group members’ uncertainty regarding issues of power and control” ( Zastrow, 2015:22), which is inherent but not an obstacle to overcome. We employed an ‘I am good at’ strategy for assigning roles.

Anticipating raising the consciousness of members to realize their value and growth potential, we administered bibliotherapy. Reading and listening to literature analogous with life experiences of individuals can constructively affect their attitudes and beliefs so that they alter and reshape their thoughts and behavior for an upgraded lifestyle ( Hynes, 2019; Lenkowsky, 1987). We started this session reading out the Malayalam (native language) version of “King’s reiterated affirmation of ‘I have a dream’” speech ( Sundquist, 2009:1). Resource people including social work professors and catholic priests narrated them stories of APJ Abdul Kalam, Abraham Lincoln, Nelson Mandela, and K. R. Narayanan. The speakers explained to them their constitutional rights, government schemes and reservations for higher studies and employment, the need for performing well in academics, staying away from illicit activities, and keeping personal integrity. In addition, members recited popular poems describing the notion of ‘equality’.

At this point, group members were asked to write down their ambition and ways to achieve it.

### Mutuality–goal achievement

This stage was characterized by greater mutual acceptance, mutual aid, and self-disclosure. Group members were encouraged to work with unity to attain the immediate objective; better the academic performance that they set in the previous stage. Knowledge acquisition that activates self-determination was the opening process here, since an individual’s “knowledge, skills, and beliefs … lead to self-determination” ( Field and Hoffman, 2002:114). Towards CB, the group was provided with academic supporting grammar sessions, mathematics, and science tuition. Members good at certain topics led peer learning sessions, motivating them to focus on studies regardless of their situations. Additionally, professional training in football and swimming were given to boost their morale through sustained reciprocal interaction and interpersonal relations in the group. They benefitted from a skill training session on kitchen gardens, which covered modules on planting vegetables in limited space, selecting seeds, organic pesticides and fertilizers, and effective ways of domestic waste management. This aimed to enable them to contribute to their household by growing vegetables, which is expected to improve their connectedness with family, and to mitigate coping issues with unpleasant family environments.

### Separation–termination

The ending of a group encompasses three aspects such as relationship between members, relationship with GWR and group as a whole (( Kurland and Salmon, 1998). “A successful termination is not an emotionally traumatic event for the members” ( Gitterman and Salmon, 2009:168). We informed the group that it is about to end and encouraged members to reminisce about their group experience. GWR recapitulated the objectives and the group’s efforts to attain them. They made an affirmation on the requirement of keeping up the momentum they achieved through the intervention..

A procedural review was made using the records we kept, that included the day, date, and time of sessions, name of attendees, list of activities, name of resource person and designation, summary of the session, participants’ reflections, GWR’s reflections, and plan for the next session.

Group members prepared vignettes on their group experiences and handed them over to the GWR as a token of their gratitude.

For a statistical analysis of the effectiveness of SGW intervention, post-test using Ryff’s PSW scale was carried out among both the IG and CG. After the completion of analysis, this SGW intervention program was administered to the CG for ethical reasons that every participant has an equal right to receive the intervention. It was carried out by the same resource persons, in the same research setting.

### Fidelity of intervention

“Greater attention needs to be given to fidelity both in the development of social, psychological, and behavioral interventions and in their execution. Fidelity is imperative in all stages and phases of intervention research, design, and implementation” ( Gearing *et al.,* 2011:83). Though assessing fidelity in intervention research is important, it is rarely done ( Hardeman *et al*., 2008). In this study we assessed fidelity through three phases: delving, designing, and delivering. An intervention must be tailor-made to meet the concerns of the population; therefore, a precise exploration of their problems is crucial. We maintained fidelity using a detailed preliminary study in the first phase, and in the second phase we designed an SGW intervention with well-defined objectives, after an extensive literature review, and consulting with experts using the Delphi survey. In the third phase, we implemented the intervention for the target group with no deviation from the pre-defined protocol. All sessions were conducted under the supervision of social work experts.

## Results

The process of SGW intervention started with recruitment of participants in July, 2019, ended with final data collection and analysis by March, 2020.

### Primary outcomes

Data collected from IG and CG using Ryff’s 42-item PWB scale before and after the intervention were analyzed statically using IBM SPSS Statistics 25. The tests were conducted against the null hypothesis, *H(o): There is no significant difference between the PWB levels of participants before and after the intervention;* and alternative hypothesis, *H(a): There is a significant difference between the PWB levels of participants before and after the intervention.*

For comparing scores obtained for PWB of participants before and after the intervention, we employed a paired sample t-test for both IG (n = 20) and CG (n = 20) separately. Tests results are shown in Tables 3 and 4.

**Table 2.** Socio-demographic profile of participants.

		Intervention Group (N=20)		Comparison Group (N=20)	
		Number	%	Number	%
Age	13	6	30.0	6	30.0
	14	10	50.0	8	40.0
	15	4	20.0	4	20.0
	16	0	000.0	2	10.0
Gender	Male	11	55.0	7	35.0
	Female	9	45.0	13	65.0
Family type	Nuclear	16	80.0	14	70.0
	Joint	4	20.0	6	30.0
Religion	Hindu	10	50.0	11	55.0
	Christian	8	40.0	9	45.0
	Muslim	2	10.0	0	000.0
Caste	SC	9	45.0	7	35.0
	OBC	3	15.0	9	45.0
	General	8	40.0	4	20.0
Financial condition	APL	0	000.0	0	000.0
	BPL	20	100.0	20	100.0
Class studying	8 ^th^ Std	8	40.0	10	50.0
	9 ^th^ Std	11	55.0	10	50.0
	10 ^th^ Std	1	5.0	0	000.0
School type	Private	0	000.0	0	000.0
	Govt.	20	100.0	20	100.0
Getting sponsorship	Yes	20	100.0	20	100.0
	No	0	000.0	0	000.0
Parents’ marital status	Married	17	85.0	19	95.0
	Divorced	3	15.0	1	5.0
	Separated	0	000.0	0	000.0
	Widowed	0	000.0	0	000.0
Fathers’ education	Lower primary	0	000.0	0	000.0
	Upper primary	1	5.0	1	5.0
	High School	12	60.0	16	80.0
	Pre-degree	5	25.0	3	15.0
	Degree	2	10.0	0	000.0
	Postgraduation	0	000.0	0	000.0
Mothers’ education	Lower primary	0	000.0	0	000.0
	Upper primary	1	5.0	2	10.0
	High School	12	60.0	15	75.0
	Pre-degree	5	25.0	3	15.0
	Degree	2	10.0	0	000.0
	Postgraduation	0	000.0	0	000.0

### Interpretation

For IG ( Table 3), the *p* value obtained was <0.05 for all the six dimensions of PWB and for the total PWB; therefore, null hypothesis was rejected and alternative hypothesis was accepted. Whereas, for the CG ( Table 4), the *p* value obtained was >0.05. Therefore, we accepted the null hypothesis. Hence, it was concluded that PWB of members of IG was raised through the intervention, while CG members’ PWB remained unchanged significantly. Thus, this analysis indicates that the social group work intervention successfully raised PWB of adolescents in the IG.

**Table 3.** Comparing pre and post intervention PWB measurements for intervention group (IG).

Paired samples test-intervention group								
	Paired differences					t	df	Sig. (2-tailed)
	Mean	Std. deviation	Std. error mean	95% confidence interval of the difference				
				Lower	Upper			
Autonomy	−20.10000	1.68273	0.37627	−20.88754	−19.31246	−53.419	19	0.000
Environmental mastery	−20.20000	2.58742	0.57856	−21.41095	−18.98905	−34.914	19	0.000
Personal growth	−21.05000	3.70597	0.82868	−22.78445	−19.31555	−25.402	19	0.000
Positive relations	−21.60000	2.89100	0.64645	−22.95303	−20.24697	−33.413	19	0.000
Purpose in life	−21.45000	3.51650	0.78631	−23.09577	−19.80423	−27.279	19	0.000
Self-acceptance	−21.70000	3.58506	0.80164	−23.37786	−20.02214	−27.069	19	0.000
Psychological well-being	−126.10000	12.12609	2.71148	−131.77519	−120.42481	−46.506	19	0.000

**Table 4.** Comparing pre and post intervention PWB measurements for the control group (CG).

Paired samples test-control group								
	Paired differences					t	df	Sig. (2-tailed)
	Mean	Std. deviation	Std. error mean	95% confidence interval of the difference				
				Lower	Upper			
Autonomy	−0.40000	1.75919	0.39337	−1.22332	0.42332	−1.017	19	0.322
Environmental mastery	−0.35000	1.18210	0.26433	−0.90324	0.20324	−1.324	19	0.201
Positive relations	−0.10000	1.07115	0.23952	−0.60131	0.40131	−0.418	19	0.681
Purpose in life	−0.30000	1.08094	0.24170	−0.80589	0.20589	−1.241	19	0.230
Self-acceptance	0.20000	1.96281	0.43890	−0.71862	1.11862	0.456	19	0.654
Personal growth	0.00000	1.89181	0.42302	−0.88539	0.88539	0.000	19	1.000
Psychological well-being	−0.95000	4.05845	0.90750	−2.84942	0.94942	−1.047	19	0.308

### Qualitative evaluation and outcomes

Content analysis ( Drisko and Maschi, 2016) of the preliminary case studies unfurled the needs and concerns of adolescents in the CSP. The risk factors identified were: low family income, stressful life events, addiction of parents, parental conflicts, single parenting, absence of social support, and absence of self-esteem and aspirations.

He always hangs out with his friends in the neighborhood. He doesn’t like studying … none in his gang is good at studies … I wish if he could study well and support our family … if things are going like this, he will become a daily wager like me … who will make him understand? (C1:M)

My husband doesn’t provide for us. Whatever he earns, he spends in the bar … he abuses us … she doesn’t even look at him … now a days she is not talking much to me also … she scores very less in exams … she doesn’t even bother that … I don’t know what would be her future … (C3: M)

I am a daily wager … I can’t spend much for her studies because I need to save something for her wedding … she is also not that interested in studies, but ready to marry someone … (C5: F)

I can’t dream high because we are poor. I just want to get through 10 ^th^ grade exam to get a driving license … I don’t have friends in my neighborhood. They all study in private schools, but I go to a government school. (C8: B)

My father is no more and my mother is doing tailoring. I help her in that and I will be continuing with that … so, there is no point in studying more … (C10: G)

Based on these results, we postulated that adolescents in the CSP need psychological support, family support, and community support along with financial support, and this study developed a program for providing them an enhanced PSW.

A concomitant analysis of qualitative data collected through the intervention process enabled refining of the intervention.

The first session was characterized with members’ anxiety about an unknown situation. Based on this observation, more group games included in the next two sessions in order to reduce their anxiety and to improve group interaction. Further, the objectives of the intervention were explained well to make participants understand that the intervention is aiming at their development.

After the first session I found a few members skeptical about the goals of the group. I overheard one boy saying that they are dubious goals. Their mind is bounded with unattainability of life goals … I was confused that what was hindering a few, particularly girls, from interacting well, fear or timidity or something else? (GWR reflections)

There were two members good at singing and they sang their favorite songs. They both were assigned to start the following sessions with a song alternatively.

GWR: You’re an excellent singer … why do you want to become a driver?

Member (M): I am not learning music … it’s a very competitive field … it will be a big failure, if I choose singing as my profession.

GWR: Not just music lessons, but talent matters in singing … You’ve got that talent, that you must cherish …

M: Ok … but … no one supports me at home …

GWR: (To the group) What you want him to be? A driver or singer?

Group (G): A singer.

Another M: He is the winner in district level youth festival.

(GWR starts clapping and the whole group follows)

GWR: We all are your supporters.

M: (Smiling) Thank you.

GWR: Now … what do you choose? To be a Driver or Singer?

M: Both … I will try for chances to sing … but I am skeptical if it can provide me enough …

… … ……………………………………… … ..

Another member was good at drawing, and he was assigned to create the welcome board for the resource people. Establishing specific roles and developing mutual trust, the group advanced further.

In the ‘goal setting’ session, all the participants shared the goals that they wrote down in the group with a lessened inhibition. Nonetheless, all were encouraging others well with cheers and applause.

An analysis of vignettes provided a real picture about the impact of this SGW intervention program.

I am very thankful to (GWR’s name). I am attending such a helpful program for the first time in my life. I came to know that I am not alone, there are many children like me. I understood the need for studying well using the aid from CSP. I want to get a decent job to support my family and I hope to end our struggles (Group member 2).

I got real friends. They won’t look down on me. Now I feel that I am a valued person. I want to study well and reach a good position (Group member 13)

This qualitative outcome in congruence with the results of statistical tests that showed a significant growth in the PWB of participants, proved the effectiveness of SGW intervention in raising PWB of under privileged adolescents.

### Follow-up

Thematic analysis of qualitative data collected from IG members’ parents, and their school teachers post intervention confirmed the secondary outcomes of SGW. Themes identified were gaining confidence to open up, articulating aspirations, showing academic progress, reducing truancy and involving in family matters.

I had never seen him before talking to others in the class, but now he interacts … not much, but … and he answers my questions too … (FGD1:02).

They are regular to school now … and they are showing some interest in studies (FGD3:04).

Now a days he goes to school every day … I don’t see him hanging out with friends … (M1).

She helps me in doing chores … she was so lazy before (M3).

These results ascertained the effectiveness of SGW in meeting the projected secondary outcomes. Being the best fit for raising PWB of CSP enrollees in the social and cultural context of research setting, these results defined the external validity and generalizability of the SGW developed and administered.

## Discussion

The outcome of this study supports the view that SGW is instrumental in equipping adolescents with “a sense of competence and a feeling of hope” ( Malekoff, 2015:20), for setting and reaching goals that upgrade their social status. This SGW intervention study not only provided participants a platform, but empowered them also to share their experiences, voice their views, and to propose a way to a better future as a transformation affords them aspirations.

In fact, for the realization of well-being and social mobility of underprivileged adolescents, we need scientific social interventions addressing the “intergenerational perpetuation of inequalities” ( Hopenhayn, 2012:1). In congruence with UNICEF guidelines for Adolescent Development and Participation ( Adolescent development and participation | UNICEF India), India has several policies and programs for the empowerment of ‘the second decade’. Nonetheless, the top-down policies have to be complemented by grassroot level interventions with a strong theoretical base and a clear conceptual framework ( Crescenzi and Rodríguez-Pose, 2011).

Considering the positive outcome this intervention research achieved, we recommend the SGW intervention to adolescents in similar contexts, with needed refining. Although this study took all possible efforts to consider expert opinions in all stages, it was limited to the professors in South India. Consulting with experts all around the world may draw wide range of opinions contributing to the fine-tuning of intervention. Moreover, this study endorses use of qualitative data in all stages of an intervention research.

While extending the literature and contributing to the research method repertoire, this article proposes a conceptual framework for the CSP, for the sustainability of well-being that participants acquired through the SGW intervention. CSPs must be inclusive to reach the over-arching goal of integrating disadvantaged adolescents into the mainstream of society. The proposed framework delineates a set of services including; a) monitoring government support systems; b) overseeing school support systems; c) providing financial aid for schooling and nutrition; d) staging community interventions; e) executing family interventions; f) conducting social groupwork; g) promoting research; h) initiating policies.

We suggest that a CSP must ensure that the adolescents enrolled in it avail the government services as well as adequate support from their schools. It must continue with sufficient financial aid to ensure accessibility to education and health care, and availability of nutritious food regardless of their socio-economic origin. Devising and carrying out community interventions is another role of CSPs to facilitate the meaningful participation of disadvantaged adolescents in their communities, with an emphasize on equity. Family interventions are crucial in making their growing environment a conducive one, whereas group interventions with adolescents are decisive in securing their PWB which triggers aspirations. Additionally, we recommend CSP to encourage scientific studies that delve into the problems of poor adolescents, formulating strategies for resolving their issues, inventing, and reinventing tools for their empowerment, and preparing policies for their social mobility *.*

**Figure 3.**
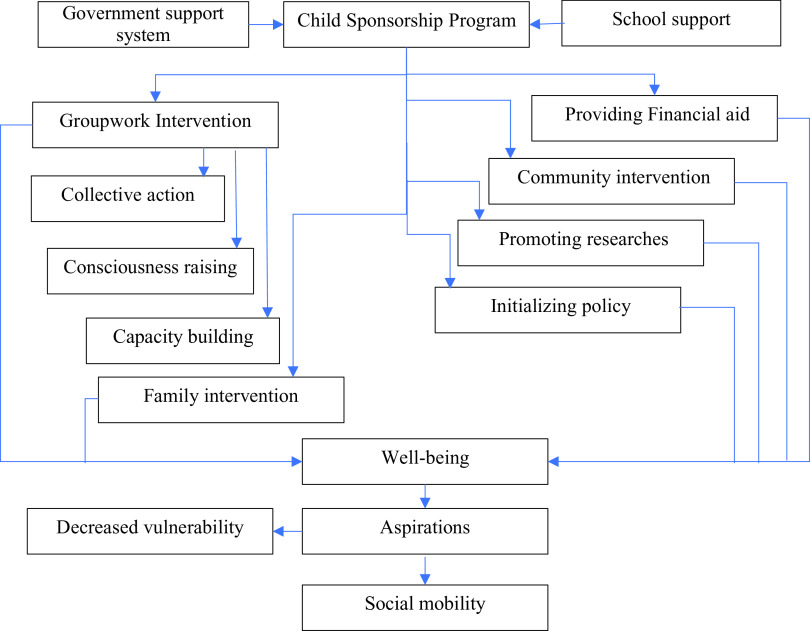
A conceptual framework for child sponsoring programs.

### Limitations of the study

In a quasi-experimental design, a potential pitfall is the imperfect matching of groups due to the heterogeneity within groups because of the variations in living environments and family histories of members ( Butler and Hickman, 2011). Another limitation is possible selection bias as this design lacks random selection of participants ( White and Sabarwal, 2014), though it offers a practical option for avoiding ethical concerns in conducting impact evaluations of an intervention with a group that is already a part of another program or agency. Besides, an interference of confounding factors is likely to occur in any experimental or interventional study. Moreover, a possible shortcoming surfaces while administering Ryff’s inventory to adolescents in that they may respond in a socially desirable way rather than their real response to the statements ( Seifert, T.A., 2005).

## Conclusion

Advocacy for marginalized adolescents is an advancing area in the realm of social work practices, which gains an additional attention apropos ‘invisibility of adolescents’ within the UN’s Sustainable Development Goals. The ‘leave no one behind’ agenda calls for research leading to policies and programs for the well-being of adolescents. This social groupwork intervention research sets forth a method for social researchers to refer to, and an effective tool for groupworkers working with adolescents in CSPs. In addition, the framework developed for CSPs is expected to be adopted by agencies to secure well-being of adolescents for capacitating them to articulate aspirations that set the stage for their transformation. Finally, we assume this research will go a long way in relegating the vicious circle of disparity, and in promoting the virtuous circle of equity.

## Data availability

### Underlying data

Harvard Dataverse: Social Groupwork for Promoting Psychological Well-being of Adolescents Enrolled in Sponsorship Programs. https://doi.org/10.7910/DVN/V6EBAX ( Joseph, 2021).

The project contains the following underlying data:

- Pre-Post Data for Well-being of Intervention Group (IG) and Comparison Group (CG).tab (SPSS file).
- Socio-economic details of the comparison group. (Anonymized details of CG).
- Socio-economic details of the Intervention group. (Anonymized details of IG).

### Extended data

Zenodo. Social Group Work for Promoting Psychological Well-being of Adolescents Enrolled in Sponsorship Programs. https://doi.org/10.5281/zenodo.4962858 ( Joseph and Karalam, 2021).

This project contains the following extended data:

- Socio-demographic details.docx (Questionnaire of socio-demographic details for participants).
- Ryff’s Psychological well-being scale.pdf (Socio-demographic questionnaire).

Data are available under the terms of the Creative Commons Attribution 4.0 International license (CC-BY 4.0).

### Reporting guidelines

Zenodo: TREND checklist for ‘Social Group Work for Promoting Psychological Well-being of Adolescents Enrolled in Sponsorship Programs’. https://doi.org/10.5281/zenodo.4962858.

Data are available under the terms of the Creative Commons Attribution 4.0 International license (CC-BY 4.0).
