# Supplementary material for: Social groupwork for promoting psychological well-being of adolescents enrolled in sponsorship programs
Source: F1000Res. 2021 Jun 30;10:520. [Version 1] doi: 10.12688/f1000research.52532.1 (PMC8276187; doi:10.12688/f1000research.52532.1)
Supplement: Peer review history

# Peer review: Reviewer response for version 1

**DOI:** 10.5256/f1000research.55827.r88776

**Published:** 2021-07-08

**Authors:** Kiran Thampi

**Affiliations:** 1 School of Social Work, Rajagiri College of Social Sciences (Autonomous), Kalamaserry, Kerala, India

I have enjoyed reading this article. The article presented an intervention study adopting a quasi-experimental, non-randomized control group design. The study explores the possibility of conducting social group work with adolescents enrolled in child sponsorship programmes to improve their psychological well-being. It is conveyed from the paper that the psychological well-being of participants in the intervention group was raised significantly due to the social group work intervention. Theoretically and methodologically, this is a well-written piece of work where the researchers were able to demonstrate considerable standards in research formulation, ethical consideration, tool selection, intervention protocols, data analysis, result presentation, and possibility for replication. Adoption of the empowerment model with strengths perspective and categorisation of group activities for IG into CA, CR, and CB is impressive. Efforts made to assess the secondary outcomes have also added to the credibility and logical connection to the findings.

 However, the following things could be taken care of/clarified by the authors to make this work more communicative to the readers.

- Research setting: Could mention how many CSP centres are there with more than 40 respondents.
- The authors could also mention, how you avoided subjective bias in selecting the 20 cases, or else, were these 20 respondents in IG?
- Figure 2: What is the attendance cut-off percentage in curtailing the number of respondents from 58 to 49 (84.48% of the registered children show up in CSP)? This could be mentioned. This is significant as the impact of CSP centres on the children is evident from this percentage and also your secondary outcomes show the impact of intervention in motivating them to be regular in their schools as well.
- Intervention: What is the time gap between the sessions you followed? This will have a considerable impact on the outcomes. This will allow practitioners to replicate the session with a similar impact.
- Tools for data collection: In the last sentence, consider avoiding apostrophe for "FGD".
- The authors have mentioned GWR in “Inclusion–orientation” in group development. You can give the expansion there as it is introduced for the first time.
- The authors may consider adding a bit on cultural context, especially for developing group norms as the SGW is much reliant on setting culturally appropriate ground rules. This will root your intervention and practitioners could be vigilant in respecting cultural implications. You may add this in the Group Development portion (four-stage model).
- Under “uncertainty-exploration” the authors mention the key personalities and their stories. Rather than mentioning their names, it would be impressive to communicate the broad areas/disciplines from which the selection of such personalities could be made. It would be good to introduce a common model template that could be adopted on the same.
- Qualitative outcomes: You have identified risk factors (themes). Since the number is 20, thematic analysis would be better. You may elaborate on the identified themes which could be supported by the respondents’ comments (from the comments you have presented now). It is always good to have literature support for those findings since you have not used your qualitative assessment to support the quantitative findings.

Is the work clearly and accurately presented and does it cite the current literature?

Yes

If applicable, is the statistical analysis and its interpretation appropriate?

Yes

Are all the source data underlying the results available to ensure full reproducibility?

Yes

Is the study design appropriate and is the work technically sound?

Yes

Are the conclusions drawn adequately supported by the results?

Yes

Are sufficient details of methods and analysis provided to allow replication by others?

Yes

Reviewer Expertise:

Mental Health, International Social Work, social work education, Development communication, participatory community appraisal, NGO management

I confirm that I have read this submission and believe that I have an appropriate level of expertise to confirm that it is of an acceptable scientific standard.

# Peer review: Reviewer response for version 1

**DOI:** 10.5256/f1000research.55827.r88773

**Published:** 2021-07-08

**Authors:** Jed W. Metzger

**Affiliations:** 1 Department of Social Work, Nazareth College, Rochester, NY, USA

Strong research. This study should be indexed.

 One question I have is why were the adolescents with a mental health concern not included in the sample? With mental health being such a key driver, and so much more common today, it is hard to understand the exclusion. I would like a little more information about the actual intervention.

 The study is mostly quantitative, but perhaps the qualitative data is richer given the small sample size.

 Not entirely clear where the group that did not receive the intervention came from.

 In a few places (the first sentence in the Abstract for instance) the writing is so dense that it could be reworked for flow.

Is the work clearly and accurately presented and does it cite the current literature?

Yes

If applicable, is the statistical analysis and its interpretation appropriate?

Yes

Are all the source data underlying the results available to ensure full reproducibility?

Yes

Is the study design appropriate and is the work technically sound?

Yes

Are the conclusions drawn adequately supported by the results?

Yes

Are sufficient details of methods and analysis provided to allow replication by others?

Partly

Reviewer Expertise:

Clinical intervention with youth

I confirm that I have read this submission and believe that I have an appropriate level of expertise to confirm that it is of an acceptable scientific standard.
